# Identification of iGb3 and iGb4 in melanoma B16F10-Nex2 cells and the iNKT cell-mediated antitumor effect of dendritic cells primed with iGb3

**DOI:** 10.1186/1476-4598-8-116

**Published:** 2009-12-07

**Authors:** Bianca R Dias, Elaine G Rodrigues, Leonardo Nimrichter, Ernesto S Nakayasu, Igor C Almeida, Luiz R Travassos

**Affiliations:** 1 Experimental Oncology Unit (UNONEX), Department of Microbiology, Immunology and Parasitology, Universidade Federal de São Paulo, São Paulo, Brazil; 2 Instituto de Microbiologia Prof Paulo de Góes, Universidade Federal do Rio de Janeiro, Rio de Janeiro, Brazil; 3 The Border Biomedical Research Center, Department of Biological Sciences, University of Texas at El Paso, Texas, USA

## Abstract

**Background:**

CD1d-restricted iNKT cells are protective against murine melanoma B16F10-Nex2 growing subcutaneously in syngeneic C57Bl/6 mice as inferred from the fast tumor development in CD1d-KO in comparison with wild type animals. CD1d glycoproteins are related to the class I MHC molecules, and are involved in the presentation, particularly by dentritic cells (DC), of lipid antigens to iNKT cells. In the present work we attempted to identify the endogenous lipid mediator expressed in melanoma cells inducing such immunesurveillance response and study the possibility of protecting animals challenged with tumor cells with lipid-primed DC.

**Results:**

Crude cytosolic and membrane fractions from *in vivo *growing melanoma contained iNKT-stimulating substances. Lipids were then extracted from these cells and one of the fractions (i.e. F3A) was shown to prime bone marrow-derived dendritic cells (BMDC) to stimulate iNKT murine hybridoma (DN32D3) cells to produce IL-2. The active fraction was analyzed by electrospray ionization-mass spectrometry (ESI-LIT-MS) and both iGb3 and iGb4 were identified along with GM3. When iGb3 was incubated with BMDC and tested with DN32D3 cells, IL-2 was equally produced indicating iNKT cell activation. GM3 consistently inhibited this response. To assess the antitumor response-induced by iGb3, a cytotoxicity assay *in vitro *was used with [^3^H]-thymidine labeled B16F10-Nex2 cells. At target/effector (iGb3-activated iNKT) cell ratio of 100^-1^-100^-4 ^tumor cell lysis was shown. The antitumor activity *in vivo *was tested in mice challenged i.v. with B16F10-Nex2 cells and treated with iGb3- or α-galactosylceramide-primed DCs. A 4-fold lower tumor load in the lungs was observed with either treatment.

**Conclusion:**

Our results show the expression of globo and isoglobohexosylceramides in murine melanoma B16F10-Nex2. The expression of iGb3 and its precursor, iGb4, on tumor cells may prime an effective iNKT cell-dependent antitumor response, modulated negatively by GM3 which is also produced in these cells. iGb3-primed BMDC exerted a significant iNKT cell-mediated anti-tumor activity in mice challenged with melanoma cells.

## Background

Murine tumors are poorly immunogenic in syngeneic mice that display the same set of major antigens and show a high degree of autologous antigen tolerance. Tumor cells grow silently in the syngeneic host until the immune system is activated either by exogenous elicitors or endogenous products of apoptotic and necrotic tumor cells. The host outcome is then determined by the imbalance between tumor cell destruction and growth, in most cases tending to the second condition, with deadly metastases. Attempts at combating the tumor cells have used proinflammatory cytokines, particularly IL-12. A gene gun-mediated skin transfection with IL-12 gene resulted in regression of established primary and metastatic syngeneic murine tumors [1]. The protective effect by IL-12 required CD8^+^, but not CD4^+ ^T cells. A tumor-specific immunological memory against a secondary tumor challenge was also observed. It is clear now that IL-12 stimulates diverse resistance mechanisms in tumors depending on the cell type, tumor microenvironment, and mouse strain [2]. It is well recognized the association of IL-12 with NK cells to produce IFN-γ, a potent antitumor agent acting directly against tumor cells [3] or upon macrophage activation. This seems to represent the immunological core of the defense mechanisms against syngeneic murine melanoma B16F10. Cui et al. [4] examined the immune cellular response in B16 melanoma, Lewis lung carcinoma, and FBL3 erythroleukemia elicited by IL-12 administration and found that Vα14-Jα18NKT cells were mainly implicated in tumor rejection.

Natural killer T (NKT) cells are subsets of lymphocytes expressing the T-cell receptor (TCR) and surface markers characteristic of NK cells such as NK1.1. Type I NKT cells express an invariant T cell receptor α-chain, Vα 14-Jα 18 in mice and Vα 24-Jα 18 in humans [5]. These cells are activated by lipid antigens presented by CD1, a molecule similar to MHC class I molecule. Type II NKT cells likewise require CD1 but have a more diverse TCR repertoire and do not recognize the most potent glycolipid known to activate NKT cells, the α-galactosylceramide (α-GalCer), derived from the marine sponge *Agelas mauritianus*. Rodents have a single CD1d gene whereas humans have 3 more CD1 antigen-presenting molecules. NKT cells have innate-like responses which may include secretion of both IFN-γ (Th-1) and IL-4 (Th-2) cytokines. Seino et al [6] showed that α-GalCer induced expansion of Vα14 NKT cells promoting inhibition of murine lung cancer. Toura et al. [7] showed that α-GalCer-pulsed dendritic cells (DC) exerted a potent antitumor cytotoxic activity against tumor metastasis mediated by NKT cells. Besides α-GalCer, many other lipids have been described to activate NKT cells such as glycosphingolipids from *Sphingomonas spp *[8], the galactosyldiacylglycerol of *Borrelia burgdorferi *[9], surface lipophosphoglycan of *Leishmania donovani *[10], and the *Mycobacterium leprae *phosphatidylinositol tetramannoside [11].

A lysosomal glycosphingolipid, isoglobotrihexosylceramide (iGb3) was found to be stimulatory in both mouse and human NKT cells [12]. Its expression in peripheral tissues could induce NKT cell activation under pathophysiological conditions such as cancer and autoimmune disease [13]. The presence of iGb4 was also detected in human thymus using mass spectrometry (MS) [14]. Mice deficient in β-hexosaminidase B (a lysosomal enzyme that converts iGb4 into iGb3) showed impaired NKT-cell development [12]. More recently, MS has been used to generate a database for glycosphingolipids from mouse thymus and among the identified species, only iGb3 is a stimulatory ligand of NKT cells [15].

In our study, we explored the anti-tumor effect of NKT cells and identified iGb3 and iGb4 as glycolipids from murine melanoma B16F10- Nex2 cells, the former being able to activate NKT DN32D3 hybridoma cells to exert antitumor responses *in vitro *and *in vivo*.

## Methods

### Reagents

Isoglobotri- and tetrahexosylceramide (iGb3 and iGb4) were purchased from Alexis - Biochemical, PA and monosialoganglioside 3 (GM3) from Matreya, PA. The α-galactosylceramide (α-GalCer) was provided by Dapeng Zhou, MD Anderson Cancer Center, Houston, TX. Solvents and reagents used for high performance thin layer chromatography (HPTLC) were purchased from Merck, Germany: methanol, chloroform, precoated Silica Gel-60 HPTLC plates (10 × 10 cm); orcinol reagent (0.5 g orcinol in 100 ml 3 M sulfuric acid); resorcinol reagent (200 mg resorcinol in 80 ml HCl and 0.25 mL 0.1 M copper sulfate, and water to 100 ml); iodine.

### Mice

Inbred male 6-8 week-old C57Bl/6 mice (WT) were purchased from the Center for Development of Experimental Models, Federal University of São Paulo (UNIFESP). CD1d knockout (KO) mice of C57Bl/6 genetic background were provided by Ricardo T. Gazzinelli (Rene Rachou Institute, Fiocruz, Belo Horizonte Brazil). KO animals were bred and maintained at the Animal Facility of Cellular Biology Division/Experimental Oncology Unit, UNIFESP. All animals were maintained in spf (specific pathogen-free) conditions, and were used in accordance with Animal Ethics Committee of UNIFESP, protocol no. 01561/2004.

### Cell Lines and Culture Conditions

The murine melanoma B16F10-Nex2 was subcloned at the Experimental Oncology Unit (UNONEX) from the cell line B16F10 obtained from Ludwig Institute for Cancer Research (São Paulo, Brazil). CD1d-transfected B6 mouse C57SV fibroblasts [16] and the NKT DN32D3 hybridoma [17] were provided by Ricardo T. Gazzinelli (Fiocruz, Belo Horizonte, Brazil). Hybridoma cells were maintained in 50% RPMI-1640 medium and 50% α-MEM medium (both from GIBCO), supplemented with 5% heat-inactivated fetal calf serum (FCS), 2 mM L-glutamine, 100 U/ml penicillin/streptomycin and 50 μM 2-mercaptoethanol (all reagents from Invitrogen, Brazil). Other cell lines were maintained in RPMI-1640 medium supplemented with 10% FBS, 10 mM N-(2-hydroxyethyl)-piperazine-N'-2-ethanesulphonic acid (HEPES), 24 mM sodium bicarbonate (both from Sigma, St.Louis, MO), 40 mg/ml gentamycin (Schering-Plough, São Paulo, Brazil), pH 7.2. All cells were maintained at 37°C in humidified atmosphere containing 5% CO_2_.

### Dendritic cell differentiation from murine bone marrow progenitors (BMDC)

Femurs from C57Bl/6 WT and KO mice were collected, muscular tissue removed, and bones were washed sequentially with 70% ethanol, iodide alcohol and PBS supplemented with gentamycin 40 mg/ml, penicillin 100 U/ml, streptomycin 100 μg/ml (PBS gen/pen/str). After cutting both ends of the femurs, the bone marrow was flushed out with PBS gen/pen/str and the cells were centrifuged at 1,200 rpm for 5 min. The cells were suspended in 10 ml/femur of RPMI 1640 supplemented with 10% of FCS, non-essential aminoacids (50×), 50 μM 2-mercaptoethanol (all from Gibco, Minneapolis, MN), 30 ng/ml murine rGM-CSF, and 10 ng/ml murine rIL-4 (both cytokines from PeproTech, Mexico) and placed in Petri tissue culture dishes (100 mm, Corning, NY). The cultures were fed with complete medium every 3 d after gently swirling the plates and replacing 80% of the spent medium. After 6-7 days of culture, large numbers of typical dendritic cells were released. These cells were thereafter pulsed with α-GalCer, iGb3 or lipid fractions extracted from B16F10-Nex2 tumor cells.

### Phenotypic analysis of mature BMDC

Bone marrow-derived dendritic cells (BMDC) were plated in 96-well plates (TPP, Switzerland) and stimulated with 200 ng/ml LPS (Sigma, São Paulo, Brazil) or 200 ng/ml LPS associated to IFN-γ 100 U (PeproTech, Mexico) for 24 h. Cells were harvested and incubated with normal murine serum for 30 min on ice to block Fc receptors and inhibit nonspecific staining. After 2 rounds of PBS washing, 5 × 10^5 ^to 10^6 ^cells/sample were incubated for 1 h on ice with combinations of the following antibodies (1:30 dilution), purchased from Pharmingen (San Diego, CA): anti-CD86 (B7.2)-PE, anti-MHC-II-FITC, anti-CD1d-FITC, biotinylated anti-CD11c (revealed with streptavidin-PE). Surface fluorescence was measured on a FACS Calibur flow cytometer (BD Biosciences, São Paulo, Brazil), and data analyzed by the CellQuest Pro software (Becton Dickinson, San Jose, CA).

### Subcellular fractions from in vivo grown murine melanoma B16F10-Nex2 cells

**Figure 1:**
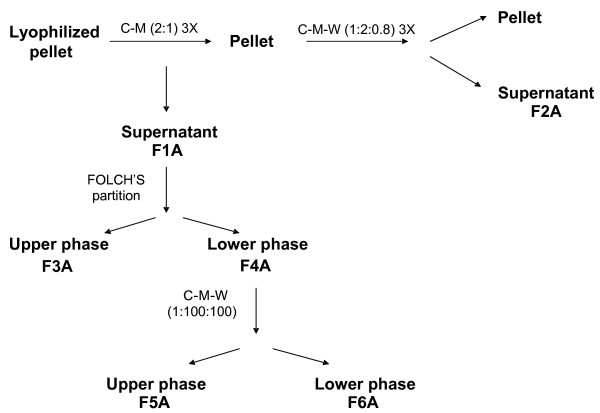
**Scheme for lipid extraction of B16F10-Nex2 murine melanoma**. Six fractions (F1A to F6A) were obtained from lyophilized subcutaneously grown tumors.

WT mice were inoculated subcutaneously with 5 × 10^4 ^B16F10-Nex2 murine melanoma cells. Tumor volumes were measured every 3 days using a caliper and the formula V = 0.52 (D × d^2^), where D and d are long and short tumor diameters respectively. Tumors were excised at 1,500 mm^3 ^and maintained frozen at -80°C. *Cytosolic *and *membrane *fractions were obtained by freezing-thawing tumors in liquid nitrogen. Lysed tumors were suspended in 50 ml PBS, filtered in nylon mesh and centrifuged at 441 *g *for 5 min for debris removal. The supernatant was then submitted to ultracentrifugation at 100,000 *g *for 90 min. The *cytosolic *fraction is represented by the resulting supernatant, and the pellet resuspended in RPMI 1640 medium supplemented with 10% FCS and 2% of DMSO, contained the *membrane *fraction. Total protein was measured in both fractions, as described [18]. To isolate lipid fractions from B16F10-Nex2 tumor cells, frozen tumors were lyophilized and 200 mg (dry weight) were extracted 3× with chloroform/methanol (2:1, v/v). The suspension was centrifuged in glass tubes (Pyrex) for 30 minutes at 1,764 *g*, the supernatant was collected, dried in nitrogen stream and this fraction was named *F1A*. The pellet was re-extracted 3× with chloroform/methanol/water (1:2:0.8, v/v/v) and the material was processed as described for *F1A*. This fraction was named *F2A*. From *F1A*, we obtained 2 more fractions by Folch's partition [19]. The upper aqueous phase was named *F3A *and the lower phase *F4A*. *F4A *was extracted with chloroform/methanol/water (1:100:100, v/v/v) and centrifuged in glass tubes for 30 minutes at 1,764 *g *and 4°C, generating two more fractions, *F5A *(upper phase) and *F6A *(lower phase). All 6 fractions were solubilized in chloroform/methanol (1:1, v/v) and desalted on C18 Sep-Pac Plus Columns (Waters Corporation, Millford, MA), according to the manufacturer's instructions. All supernatants were dried under nitrogen stream and stocked in a glass desiccator before use (Fig. 1).

### HPTLC resolution of fractions F3A and F4A

Fractions F3A and F4A were dissolved in chloroform/methanol (1:1, v/v), sonicated on a water bath for 30 seconds, and 1 mg/ml (dry weight) was applied on HPTLC silica plates (Sigma). The mobile phase was chloroform/methanol/water (60: 35: 8, v/v/v). Run HPTLC plates were revealed with orcinol reagent (for hexose detection), resorcinol-HCL reagent (for sialyl-containing carbohydrates) and iodine vapor (for total lipids). Ganglioside GM3 and isogloboside iGb3 (5 μg/ml), used as standards, were visualized with these reagents, and R_F _values determined. Tumor F3A and F4A fractions were chromatographed and at the R_F _values corresponding to GM3 and iGb3 the silica was scraped off and extracted with chloroform: methanol (1:1, v/v), then centrifuged for 30 min at 1,764 *g*. The supernatant was collected and dried under nitrogen stream. Preparative fractions isolated from F3A and F4A chromatographies were called *iF3A *and *iF4A*, respectively. The iF3A and iF4A fractions were dissolved in chloroform: methanol (1:1, v/v), sonicated for 30 seconds, resolved on silica gel 60 Å TLC plates (10 × 10 cm, 0.25 mm, Merck) and compared with GM3 and iGb3 standards.

### In vitro stimulation of NKT DN32D3 hybridoma cells with α-GalCer, iGb3 and fractions of tumor B16F10-Nex2

On the 6^th ^day of *ex-vivo *culture, BMDCs were stimulated with α-GalCer (0.01-10 ng/ml) in complete medium (RPMI 1640 supplemented with 10% FCS, 50 μM 2-mercaptoethanol, 2 mM glutamine, gentamycin 40 mg/ml, penicillin 100 U/ml, streptomycin 100 μg/ml, and 10 mM HEPES) for 24 h. The NKT hybridoma DN32D3 (5 × 10^4^) cells were co-cultured with 5 × 10^4 ^BMDC, stimulated or not, in 96-well flat bottom microplates (TPP) in triplicates, in a total volume of 200 μl/well. After 18 h at 37°C and 5% CO_2_, supernatants were collected for IL-2 measurement by ELISA. The same method was applied for stimulation of BMDCs with iGb3 (0.5-100 μg/ml), cytosolic fraction (0.008-3.41 mg protein/ml), membrane fraction (0.007-1.1 mg protein/ml), F3A fraction (15-1,000 μg/ml), iF3A fraction (1-100 μg/ml) and iF4A fraction (5-50 μg/ml). A few experiments were also carried out with CD1d-transfected fibroblasts as antigen-presenting cells, stimulated as described for BMDCs.

### Measurement of IL-2 by ELISA

Enzyme-linked immunosorbent assay (ELISA) plates were coated with 2 μg of murine anti-IL-2 monoclonal antibody in 50 μl binding buffer (0.1 M Na_2_HPO_4_, pH 9.0) and incubated overnight at room temperature. After 3 rounds of washings (with PBS containing 0.05% Tween-20), plates were blocked for 2 h at room temperature, with PBS containing 1% bovine serum albumin and 0.05% Tween-20. After washing, 50 μl/well of murine recombinant IL-2 at 0.032-4 ng/ml diluted in PBS-1% BSA, or 100 μl/well of culture supernatant were added and incubated overnight at 4°C. Plates were washed and biotin-conjugated murine anti-IL-2 monoclonal antibody (50 ng/100 μl/well) was added, following incubation for 2 h at room temperature, washing and further incubation for 1 h at room temperature with 50 μl/well of HRP-streptavidin (1:1000, diluted in PBS - 1% BSA). Reaction was revealed by addition of 50 μl/well of 5 ml citrate-phosphate buffer pH 5.5, 2 ml water, 2 mg OPD and 10 μl H_2_O_2_. A solution of 4N H_2_SO_4 _(50 μl/well) was used to terminate the reaction. Absorbance was measured at 490 nm. All reagents were purchased from Pharmingen, San Diego, CA.

### Separation of neutral and negatively charged glycosphingolipids

Tumor lysate and F3A fraction were chromatographed in a strong anion-exchange (SAX) resin (POROS 50 HQ, Applied Biosystems, São Paulo, Brazil) for separation of neutral and charged glycosphingolipids. The column was previously washed with 4 ml methanol, 2 ml 80% acetonitrile/0.05% trifluoroacetic acid, and finally equilibrated with 10 ml methanol. Samples were diluted to a final volume of 5 ml with 100% methanol and loaded into the column. After washing with 6 ml methanol, elution was carried out with 6 ml of 250 mM ammonium acetate in 100% methanol. Neutral glycolipids were recovered in the unbound fraction, whereas the eluted fraction was composed mainly of gangliosides. All samples were dried under highly pure N_2 _stream. The eluted fraction was desalted in a 3-ml reverse phase cartridge (Discovery DSC-18, Supelco, Bellefonte, PA, USA). The cartridge was washed with 4 ml methanol, equilibrated with 5 ml deionized water, and the samples were loaded in 5 ml 0.1 M KCl. After washing with 10 ml water, the samples were eluted with 10 ml methanol and dried under highly pure N_2 _stream.

### Permethylation of glycosphingolipids

All permethylation reagents were purchased from Sigma-Aldrich, St. Louis, MO. Permethylation of glycosphingolipids was carried out as described [20]. Briefly, the samples were dissolved in 150 μL dimethylsulfoxide (DMSO), a few milligrams of powdered NaOH were added, and the mixture was vortexed vigorously. Then, 80 μL of iodomethane was added and the reaction was carried out for 1 h at room temperature in an orbital shaker. The reaction was then quenched with 2 ml water and 2 ml dichloromethane was added before the mixture was vortexed. After brief centrifugation, the aqueous phase was removed and the organic phase was washed twice with water. The final organic phase was dried under N_2 _and suspended in 200 μl pure methanol for MS as follows.

### Electrospray ionization-linear ion trap-mass spectrometry (ESI-LIT-MS) analysis

Permethylated glycosphingolipids were analyzed by electrospray ionization-linear ion trap-mass spectrometry (ESI-LIT-MS) as described by Li et al. [14,21]. Briefly, permethylated samples were loaded in static nanospray tips (New Objective) and analyzed in a linear ion-trap mass spectrometer (LTQ XL with ETD, Thermo Fisher Scientific, San Jose, CA). The spray voltage was set from 0.7 to 1.5 kV, varying according to the tip. After detecting the intact permethylated glycolipids by MS^1^, tandem fragmentation (MS^2^-MS^4^) of individual glycolipid species was carried out manually, or by total-ion mapping (TIM) of *m/z *667 (marker of Gb3 and iGb3) or *m/z *912 (marker of Gb4 and iGb4) [14,21]. The isolation window was set at 3 atomic mass units (a.m.u.) for manual fragmentation and 1 a.m.u., for TIM. The collision energy was set to 60% for either manual or TIM analysis. The spectra were annotated manually. To calculate the amount of iGb3 in fraction F3A we used the following equation: A(iGb3)_sample _= (A(iGb3)_211corr _+ A(iGb3)_371_)/[A(iGb3)_211corr _+ A(iGb3)_371 _+ (2× A(Gb3)_329_, where A(iGb3)_211corr _= A(iGb3)_predicted_/A(iGb3)_max using iGb3 standard_, where A(iGb3)_211 _and A(iGb3)_371 _are the abundance (A) of iGb3 markers *m/z *211 and *m/z *371, and A(Gb3)_329 _that of Gb3 marker *m/z *329 [21].

### In vitro cytotoxicity assay

The cytotoxic effect by activated NKT DN32D3 hybridoma cells on B16F10-Nex2 tumor cells was evaluated as described [22]. B16F10-Nex2 cells (2 × 10^5^) were incubated in 25 cm^3 ^flasks with 0.5 μCi of [^3^H] thymidine (NEN, Boston, MA) for 24 h. NKT hybridoma DN32D3 cells were activated by previous incubation with DC primed with iGb3 (20 μg/ml) or unprimed DC for 4-6 h. Activated NKT cells and [^3^H] B16F10-Nex2 cells were co-cultured for 4 h on 96-well flat-bottom microtiter plates at target/effector cell ratios of 1/12 to 1/400, in triplicates. All cells were collected in a Cell Harvester and radioactivity was measured with a β-counter. The specific cytotoxicity (% lysis) was calculated using the formula: (E-C)/E × 100, where E is the radioactivity (cpm) in the glycolipid-primed DC system and C the control radioactivity value in the unprimed DC system. Values were subtracted from the maximum radioactivity value of unchallenged labeled melanoma cells.

### In vivo experiments

WT and CD1d-KO animals (5 per group) were inoculated subcutaneously with 5 × 10^4 ^B16F10-Nex2 tumor cells, on the right flank, and tumor development was observed every 2 days for 67 days. Animals were sacrificed at maximal tumor volumes of 3 cm^3^. To verify the protective effect of activated BMDCs on the pulmonary metastatic melanoma model, WT animals were injected intravenously with 5 × 10^4 ^murine melanoma cells on day 0, and on days 2 and 4 with 5 × 10^5 ^BMDCs (from WT mice) activated *in vitro *for 24 h with 200 ng/ml α-GalCer, or 20 μg/ml iGb3. A group of WT animals was treated with BMDCs obtained from CD1d-knockout mice stimulated with 20 μg/ml iGb3. Animals were sacrificed on day 14 and the number of lung nodules was quantified using a stereomicroscope. Experiments were repeated twice.

### Statistical analysis

Experiments *in vitro *and *in vivo *were analyzed using the Student's t-test. The animal survival experiment in CD1d-KO and WT animals, was analyzed by Kaplan-Meier and logrank test. Values of *p *≤ 0.05 were considered statistically significant.

## Results

### Survival of C57Bl/6 WT and CD1d-KO mice upon challenge with melanoma cells

**Figure 2:**
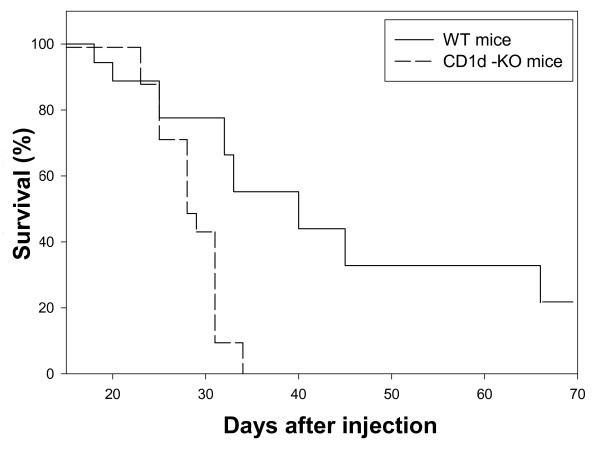
**CD1d-knockout mice allowed faster subcutaneous development of B16F10-Nex2 tumors than WT mice**. Wild type mice (--) and CD1d-knockout mice (----) were injected subcutaneously with 5 × 10^4 ^viable B16F10-Nex 2 cells. Animal survival was registered for 70 days. Mice were sacrificed when tumors reached 3,000 mm^3^. Results are representative of 4 independent experiments. p < 0.001.

Mice aged 6-8 weeks were injected subcutaneously with 5 × 10^4 ^B16F10-Nex2 melanoma cells and tumor growth was recorded every 2 days during 70 days. All CD1d-KO mice were dead or were sacrificed with tumors at the maximum size allowed after 32 days. In the WT mice, tumor development was significantly slower with 20% of mice still alive after 70 days (Fig. 2). This result suggests that CD1d-dependent effector cells (e.g. NKT cells) play an important role in anti-tumor progression in this syngeneic model.

### Activation of DN32D3 hybridoma cells

We have used NK1.1^+ ^DN32D3 cells (Vα14-Jα18/Vβ8 NKT mouse hybridoma) for stimulation *in vitro*. CD1d-transfected fibroblasts, 5 × 10^4^, plated on 96-well plate, were pulsed with different concentrations of α-galactosylceramide (α-GalCer), a classical activator of NKT cells, for 24 h. DN32D3 cells (5 × 10^4 ^cells/well) were added to a final volume of 200 μl in RPMI 1640 and co-cultured with the fibroblasts for 18 h. The culture supernatant was then collected and production of IL-2 was quantified. Alternatively, we used BMDCs for antigen presentation. BMDCs are the most efficient cell type able to present the endogenous ligand iGb3 that stimulates NKT cells (Zhou et al., 2004). These cells were cultivated with 30 ng/ml GM-CSF and 10 ng/ml IL-4 for 6 days. Half of these cells double stained for CD11c-PE and CD1d-FITC and this frequency further increased after stimulation with LPS and IFN-γ. Incubation with the glycolipid also succeeded in activating NKT cells to produce IL-2 (not shown).

### Stimulation of DN32D3 cells by cytosolic and membrane fractions of B16F10-Nex2 cells

B16F10-Nex2 cells (5 × 10^4^) were injected subcutaneously in C57Bl/6 mice and the tumor was excised when its volume reached 1,500 mm^3^. The tumor cells were lysed by freezing/thawing, centrifuged at low speed and the supernatant ultracentrifuged at 100,000 *g *to yield cytosolic and membrane fractions that were tested for stimulation of NKT cells. Both fractions were added to BMDC for 24 h and co-cultured with NKT cells.

**Figure 3:**
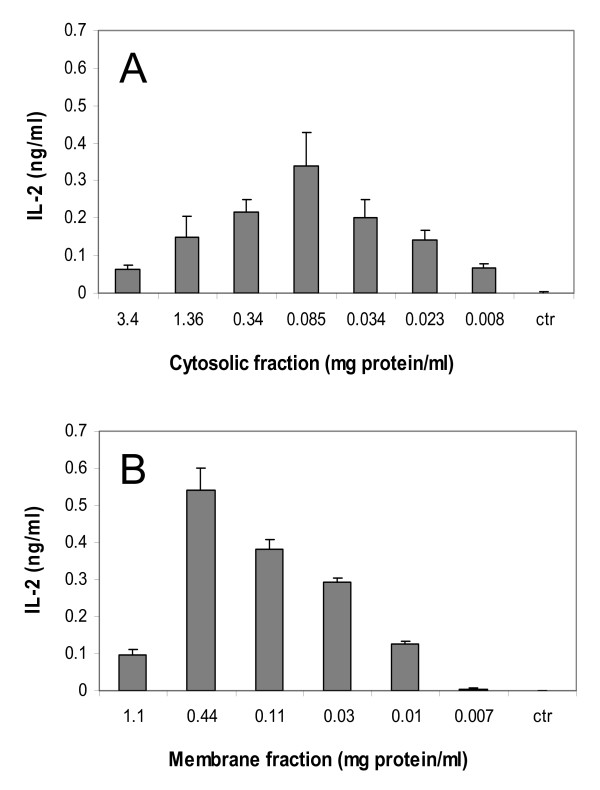
**Stimulation of DN32D3 NKT cells by cytosolic and membrane fractions of murine melanoma B16F10-Nex2**. BMDCs were pulsed with different concentrations of **(A) **Cytosolic and **(B) **Membrane fractions, both extracted from in vivo growing murine melanoma B16F10-Nex2. Primed BMDCs were co-cultured with NKT hybridoma cells as described in Material and Methods, and the IL-2 production was measured in the supernatants by ELISA. **Ctr**, DN32D3 cells stimulated with untreated BMDCs.

Stimulation of NKT cells depended on the fraction concentration (measured as mg of protein) with rather restricted amounts for optimal IL-2 production (Fig. 3).

### Lipid extraction from B16F10-Nex2 tumor cells and their ability to activate NKT cells

The lipid fractions were obtained from lyophilized subcutaneously grown melanoma cells as described in Fig. 1. All fractions desalted in SepPak C18 were tested for stimulation of DN32D3 NKT cells. At 250 μg/ml the F3A fraction stimulated NKT cells to produce 1 ng/ml of IL-2. At lower concentrations (15-30 μg/ml) 600 pg/ml of IL-2 was secreted (not shown). It was hypothesized that both stimulatory and inhibitory lipids could be present in this fraction. As to fraction F4A, a restricted concentration, 28 μg/ml, was stimulatory with low IL-2 production. The other fractions did not give significant results and some of them inhibited the background stimulation of NKT cells (not shown).

### Putative stimulatory and inhibitory components of fractions F3A and F4A

**Figure 4:**
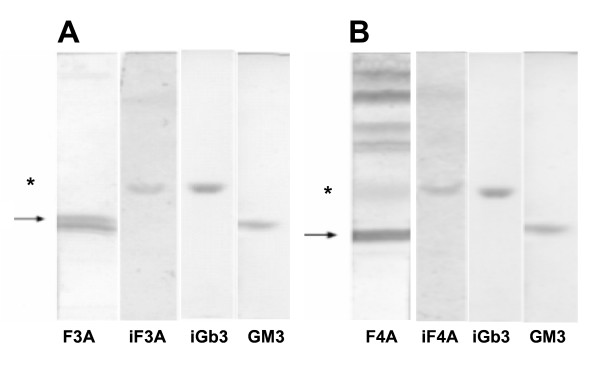
**Identification and isolation of GM3 and iGb3 fromF3A and F4A**. **A) **Fractions 20 μg, GM3 5 μg and iGb3 5 μg were chromatographed and stained with orcinol. The region corresponding to Gb3/iGb3 in an F3A preparative HPTLC was scraped off and extracted with C-M (1:1). The concentrated extract was examined by HPTLC and revealed with orcinol **(iF3A)**. **B) **The same procedure as in **(A)** was used to examine F4A and generate **iF4A**. Arrows indicate bands with R_F _corresponding to GM3 (double band) in F3A and F4A; * the same for Gb3/iGb3.

HPTLC analysis of fractions F3A and F4A showed a major component stained with orcinol (for sugars in general) and resorcinol (for sialic acid) with R_F _similar to GM3. Together with GM3, another component appeared which showed a running R_F _similar to iGb3 already reported to stimulate NKT cells [12,13]. To detect the latter, lipid fractions were resolved by preparative HPTLC developed with C-M-W (60:35:8, v/v/v), The TLC samples with R_F _corresponding to Gb3/iGb3 were scrapped off and were isolated by washing with C-M (2:1, v/v) and centrifugation, further washing (3×) of pellet with C-M (1:1, v/v) and finally 3 washes with C-M (1:2, v/v). All washes were dried and plotted on a new HPTLC plate. On Fig. 4A, a single band of R_F _similar to the iGb3 standard was stained with orcinol (iF3A). The same procedure was used for fraction F4A yielding iF4A (Fig. 4B). Fractions iF3A (5 μg/ml) and iF4A (25 μg/ml) stimulated NKT cells to produce 60 pg/ml and 150 pg/ml of IL-2, respectively. In contrast, GM3 markedly inhibited the basal production of IL-2 by unstimulated NKT cells, even at 0.035 ng/ml (not shown).

### ESI-LIT-MS analysis of fraction F3A lipid components

**Figure 5:**
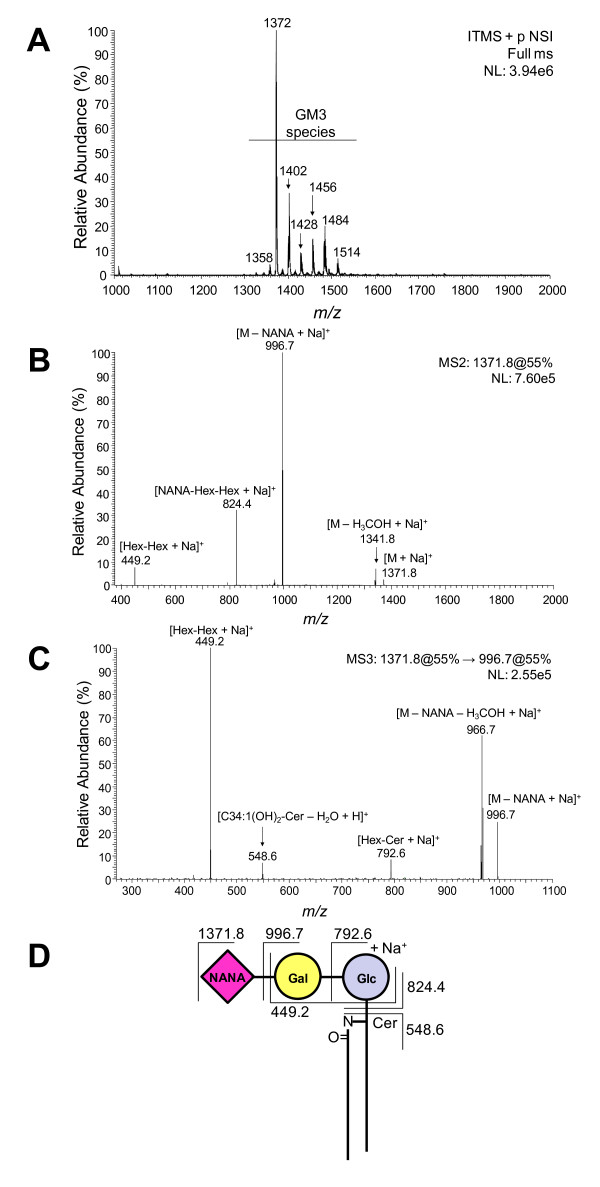
**Electrospray ionization-linear ion trap-mass spectrometry (ESI-LIT-MS) of the major glycolipid species from F3A fraction**. **A) **MS^1 ^spectrum of permethylated F3A. **B) **MS^2 ^spectrum of the singly-charged ion species ([M -H + 2 Na]^+^) at *m/z *1371.8 observed in **A. C) **MS^3 ^spectrum of the major daughter-ion species at *m/z *996.7 observed in **B. D) **Summary of key fragments observed in the MS^2^ and MS^3^ spectra of permethylated GM3 species at *m/z *1372. For simplification, the proposed GM3 structure is depicted without permethylation. *m/z*, mass to charge ratio.

Fraction F3A was permethylated and examined by ESI-LIT-MS. Several singly-charged ion species were observed at the 1300-1600 *m/z *range (Fig. 5A). These ions were compatible with GM3 species bearing *N*-acetyl- or *N*-glycolylneuraminic acid (**Δ***N*-Ac/Me-*N*-glycolyl = 30 *m/z*) and different lipid moieties. To confirm this initial prediction, the major peak at *m/z *1372 (monoisotopic mass at *m/z *1371.8) representing a singly-charged ion species with sodium adduct ([M - H + 2 Na]^+^) was subjected to MS^2 ^and MS^3 ^fragmentation (Fig. 5B-C). The MS^2 ^spectrum revealed a major daughter-ion at *m/z *996.7, resulting from the loss of *N*-acetylneuraminic acid (NANA). Also, two other ions were observed at *m/z *824.4 and 449.2, most likely corresponding to sodiated NANA-Hex-Hex and Hex-Hex fragments, respectively (Fig. 5B). To confirm the GM3 nature of *m/z *1372, the major daughter-ion species observed at *m/z *996.7 was subjected to MS^3 ^fragmentation (Fig. 5C). Two daughter-ions observed at *m/z *792.6 and 548.6, most likely corresponding to the sodiated Hex-Cer and C34:1(OH)_2_-ceramide fragments, respectively, corroborated the presence of a ceramide moiety, probably containing sphingosine (d18:1) and palmitic acid (C16:0) (Fig. 5C). Fig. 5D depicts the key fragments observed in the MS^2 ^and MS^3 ^spectra of the major GM3 species of fraction F3A.

**Figure 6:**
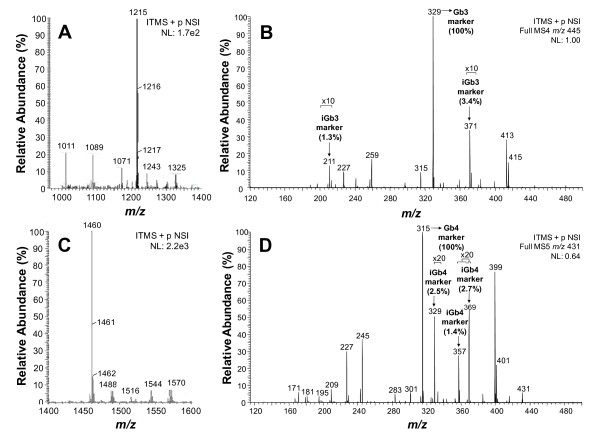
**ESI-LIT-MS analysis of GM3-depleted permethylated neutral glycolipids of F3A fraction**. **A) **Total ion-mapping of *m/z *667, marker of Gb3 and iGb3. **B) **MS^4 ^spectrum of the daughter-ion *m/z *445 obtained after MS^3 ^fragmentation (not shown) of the parent-ion at *m/z *1215 observed in **A**. The fragments at *m/z *371 (3.4%) and *m/z *211(1.3%) are typical of iGb3, whereas the fragment at *m/z *329 (100%) is a marker of Gb3. **C) **Total ion-mapping (TIM) of *m/z *912, marker of Gb4 and iGb4. **D) **MS^4 ^spectrum of the daughter-ion *m/z *431 obtained after MS^3 ^fragmentation (not shown) of the parent-ion at *m/z *1460 observed in **B **. The fragments at *m/z *329 (2.5%), *m/z *357 (1.4%), and *m/z *369 (2.7%) are characteristic of iGb4, whereas the fragment at *m/z *315 (100%) is a marker of Gb4. *m/z*, mass to charge ratio.

Due to the high amount of GM3 species, the original (non-permethylated) fraction F3A was fractionated using a SAX column to separate neutral and charged glycosphingolipids. The neutral glycosphingolipids (NGSLs) recovered in the flow-through fraction were permethylated (pMe) and analyzed by ESI-LIT-MS. Two major peaks at *m/z *1215 (pMe Galα1-3/4Galβ1-4Glcβ1-1Cer) (Fig. 6A) and at *m/z *1460 (pMe GalNacβ1-4Galα1-3/4Galβ1-4Glcβ1-1Cer) (Fig. 6C) were detected by total-ion mapping (TIM) of *m/z *667 (marker of Gb3 and iGb3) or *m/z *912 (marker of Gb4 and iGb4). The fragmentation of *m/z *1215 gave rise to *m/z *667 (pMe Galα1-3/4Galβ1-4Glcβ1-) with loss of ceramide, followed by loss of glucose (Glc) corresponding to *m/z *445 (pMe Galα1-3/4Gal-). The last fragmentation gave rise to *m/z *371 (1.3% relative abundance) and *m/z *211 (3.4% relative abundance), which are markers characteristic of iGb3, as well as the predominant peak at *m/z *329 (100% of relative abundance), a marker of Gb3 (Fig. 6B). These peaks and those at *m/z *227, 259, 315, 413, and 415 are consistent with those found by Li et al. (2008), who described the fragmentation of the nonreducing terminal disaccharide-1-ene of iGb3 and Gb3. The results showed, therefore, that F3A contains a mixture of iGb3 and Gb3. The amount of iGb3 in the sample was calculated (see equation below), corrected according to the A(iGb3)_211 _in iGb3 (expected/maximum = 100/79 = 1.27, correction factor).

**Figure 7:**
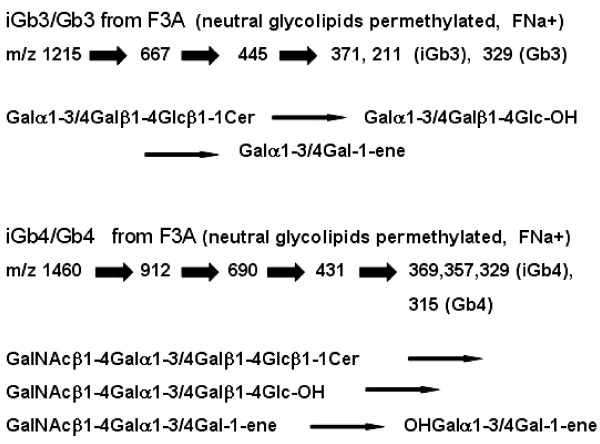
**Summary of fragmentation products in positive-ion mode ion trap-mass spectrometry of permethylated neutral glycolipids of fraction F3A**. iGb3 and Gb3 as well as iGb4 and Gb4 are recognized by the fragmentation of the disaccharide-1-ene ions (*m/z *445 and *m/z *431, respectively). F, fragment ions; FNa+, fragment with Na+ adduct.

Fragmentation of *m/z *1460 (Fig. 6D) involved primarily a loss of ceramide giving rise to *m/z *912 (pMe GalNacβ1-4Galα1-3/4Galβ1-4Glcβ1-), followed by loss of glucose, *m/z *690 (pMe GalNacβ1-4Galα1-3/4Galβ1-), and finally loss of *N*-acetyl-galactosamine, with remaining pMe disaccharide Galα1/4Gal and Galα1/3Gal (*m/z *431). Fragmentation of these generated markers of isoglobotetraosylceramide (iGb4), *m/z *329 (2.5%), *m/z *357 (1.4%) and *m/z *369 (2.7%), and a major peak characteristic of globotetraosylceramide (Gb4) at *m/z *315. Differing from the MS analysis of iGb3/Gb3, that of iGb4/Gb4 is not quantitative. A summary of the fragmentation sequences of permethylated NGSLs from fraction F3A is shown on Fig. 7.

### Stimulation of DN32D3 cells with iGb3

**Figure 8:**
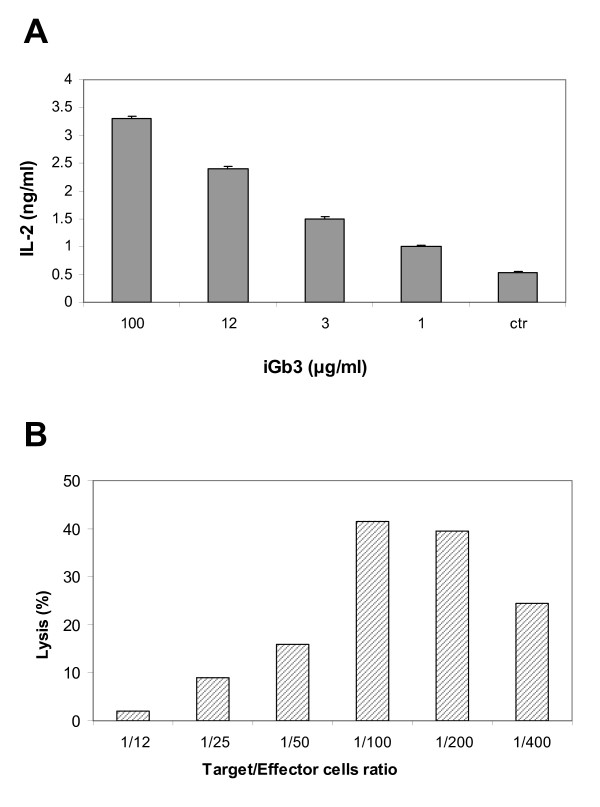
**iGb3-stimulated DN32D3 NKT cells are cytotoxic to B16F10-Nex2 melanoma cells**. **A) **BMDCs were pulsed with iGb3 for 24 h, co-cultured with DN32D3 NKT cells for 18 h, and IL-2 production was measured in the supernatant by ELISA. The results are representative of three independent experiments. **B) **BMDCs were primed with 20 μg/ml iGb3 for 24 h and co-cultured with NKT hybridoma cells for 4 h. B16F10-Nex2 cells were previously incubated with 5 μCi of [^3^H] thymidine for 24 h. Melanoma and NKT cells were co-cultured at the target/effector cell ratio indicated and the cytotoxic (lytic) effect was measured as described in Material and Methods.

Since iGb3 was identified in F3A fraction of melanoma cells and since the fraction also contained GM3 which exerted an inhibitory activity on NKT cell stimulation we tested the effect in this system of purified iGb3. On Fig. 8A it is shown that iGb3 was able to stimulate NKT cells in a dose-dependent manner being presented by BMDCs. When compared to α-GalCer, however, iGb3 was 100-fold less potent in terms of NKT cell stimulation and IL-2 production, but could still be used in microgram quantities for *in vivo *tests.

DC cells incubated with 1 μg/ml of iGb3 and analyzed by FACS showed increased expression (50%) of CD1d and slight increase of CD80 and CD86 (data not shown).

### In vitro iGb3 cytotoxicity assay in B16F10-Nex2 cells

B16F10-Nex2 cells were incubated with [^3^H] thymidine for 24 h and co-cultured for 4 h with DN32D3 NKT cells activated by BMDCs primed with iGb3 (20 μg/ml) or unprimed. The NKT (effector) cells were added at rates of 12 to 400 cells per tumor cell (target). At 100-200 iGb3-activated NKT effector cells to 1 target cell ratio there was a net 40% lysis of the tumor cells (Fig. 8B) after subtraction of the control lysis with no exogenous activation of NKT cells.

### In vivo anti-tumor protection of BMDC primed with iGb3 and α-GalCer

**Figure 9:**
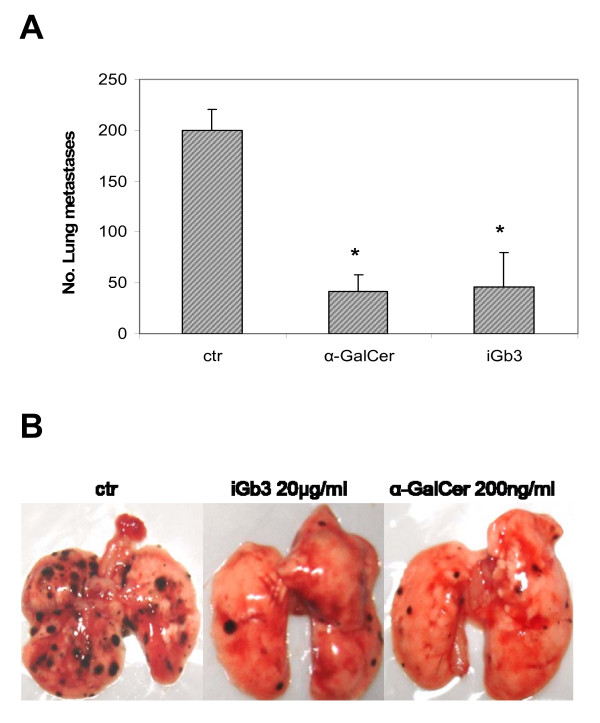
***In vivo *antitumor effects of α-GalCer and iGb3**. **A) ***In vivo *protection by α-GalCer (200 ng/mL) and iGb3 (20 μg/ml) against lung colonization by B16F10-Nex2 melanoma cells (1 × 10^5^) injected i.v. in C57Bl/6 mice (5 animals/group). Glycolipid-primed BMDCs or unprimed BMDCs were administered on days 2 and 4 after challenge. **Ctr**, control, unprimed naïve BMDCs; **B) **Lungs representative of animals treated with unprimed and glycolipid-primed BMDCs after 13 days of tumor challenge. The experiments are representative of at least 2 independent experiments. *p < 0.05, compared to the control.

Effective treatment of mice challenged intravenously with B16F10-Nex2 cells was investigated using BMDCs primed with iGb3 (20 μg/ml) and α-GalCer (200 ng/ml). Mice were injected with 5 × 10^4 ^melanoma cells/100 μl/animal and treated on days 2 and 4 with BMDCs primed with glycolipids. On Fig. 9A we show that animals treated with α-GalCer and iGb3-primed BMDCs had 4-fold fewer nodules than animals treated with unprimed DC. Clearly on Fig. 9B we show that lungs of animals treated with BMDC-glycolipids have very few nodules when compared to the control animals. These results show that iGb3 similarly with α-GalCer can display anti-tumor activity when presented by BMDCs. That the anti-tumor effect depended on cytotoxic NKT cells is inferred from the inability of iGb3-treated BMDCs from CD1d-KO mice to show any protective activity (not shown).

## Discussion

NKT cells are at the edge of innate and adaptive immunity, and have important roles in infectious diseases, autoimmunity and cancer modulating activity, either promoting or inhibiting tumor development. Clearly different subtypes, time of activation, soluble or cell-bound ligands are involved in these contradictory effects. Generally, anti-tumor activities are linked to direct cytotoxicity of type I NKT cells expressing perforin, FasL, TRAIL, but mainly IFN-γ that activates other immune cells such as DCs, NK cells and T cells [23]. These cells also produce IL-4 upon activation with α-GalCer in the mouse, but after initial stimulation, NKT cells are polarized for the production of IL-4 with simultaneous increase in serum IgE levels, thus modulating the immune response toward a Th2 phenotype [24]. Type II NKT cells are regulatory cells that suppress CD8^+ ^CTL-mediated antitumor activities. In a model of tumor recurrence the CD8^+ ^T cell-mediated immunosurveillance was suppressed by IL-13 producing CD1d-restricted CD4^+ ^T cells [25]. We also found evidence that regulating NKT cells could be involved in the immune response against B16F10-Nex2 melanoma in the mouse by the protective effect exerted by the neutralization of IL-13 using an IL-13Rα2-Fc chimera, enhanced by IL-12 [26].

In the present work, it is evident that type I NKT cells (invariant or iNKT) play an important role in the protection against B16F10-Nex2 melanoma cells based on the enhanced tumor progression in CD1d-KO animals. At a limited density of tumor cells, WT mice showed increased survival and after 70 days of tumor challenge, 20% of animals were still alive. IFN-γ-producing iNKT cells seemingly play a role in anti-tumor protection by activating other cytotoxic lymphocytes mainly through Th1 cytokine cascades. Rejection of tumor was also observed when the activation of iNKT cells by yet unidentified tumor-derived ligands fostered a CD4^+ ^and CD8^+ ^adaptive immune response [27].

For a CD1d-restricted protective response of iNKT cells in the B16F10-Nex2 system, endogenous lipid components of tumor cells should be recognized and we hypothesized that they could be glycosphingolipids expressed on tumor cells, particularly iGb3. Apparently, Vα14 TCR in iNKT cells befits the foreign archetype ligand α-GalCer and the recently described endogenous iGb3 [12,13] by adopting two different conformations [28]. Although iGb3 has been described as a candidate for the main endogenous ligand of iNKT cells the biochemical fractionation of natural antigens is hampered by their low abundance in the starting materials used. In fact, iGb3 was not found in mouse or human thymus or DCs using a sensitive HPLC assay [29]. Another report claimed that humans lack iGb3 due to the absence of a functional iGb3 synthase [30]. It would seem, based on these reports, that iGb3 is unlikely to be a physiologically relevant NKT cell-selecting ligand in mouse and humans. In contrast to these reports, however, isoglobo and globo series tetraglycosylceramides have recently been identified in human thymus, and iGb3 and iGb4 have been identified in the mouse thymus using the more sensitive ESI-LIT-MS [14,15,21]. As pointed out by Li et al. [21] in relation to the pseudogene hypothesis of human iGb3 synthase, mRNA transcripts for iGb3 synthase in human thymus have otherwise been recently detected and a full-length cDNA has been cloned (unpublished data).

In B16F10-Nex2 cells iNKT stimulating components were present in the cytoplasm and cell membrane fractions. Orcinol- and resorcinol-reacting glycolipids were extracted from the subcutaneously grafted tumor with a predominance of GM3 as expected for murine melanoma cells. The fractions obtained after Folch's partition also showed the presence of a glycolipid with the same R_F _of iGb3 standard in HPTLC. The amount was quite small but a preparative chromatography succeeded in the enrichment of this species. Both GM3 and neutral glycolipids had their presence confirmed in the permethylated form by ESI-MS. Fragmentation of a predominant peak from GM3 by MS^2 ^and MS^3 ^showed the presence of *N*-acetylneuraminic acid and C34:1-(OH)_2_-ceramide, most likely containing sphingosine (d18:1) and palmitic acid (C16:0). Other species with different lipid moieties were also shown in permethylated derivatives and are compatible with the double band in HPTLC, a common characteristic of GM3 [31]. The disproportionate amount of GM3 in relation to iGb3 may explain the low NKT cell stimulation of melanoma lipid fractions containing a mixture of these glycolipids. Indeed, GM3 showed inhibitory activity of NKT cells [32] and it is generally accepted that gangliosides are immunosuppressive cell surface molecules often present in high concentrations in tumor cells. These molecules may inhibit the immune response that is implicated in tumor rejection. B16 murine melanoma sublines with pharmacologically decreased concentration of gangliosides produced fewer tumors in mice than untreated cells [33]. Moreover, a GM3-conjugated vaccine induced anti-tumor activity against B16 melanoma *in vitro *and *in vivo*, and this effect was antibody-dependent [34]. Here, we observed that with nonprimed BMDC, the background production of IL-2 by NKT cells was completely inhibited by GM3.

Even considering the low amounts of isoglobotri- and isotetrahexosyl ceramides (iGb3 and iGb4) in mammalian cells they were identified in B16F10-Nex2 melanoma along with predominant globotrihexosyl and globotetrahexosyl ceramide (Gb3 and Gb4) components by ion trap mass spectrometry. Functionally, only iGb3 can stimulate NKT cells [12] but iGb4 is converted to iGb3 in cells expressing β-hexosaminidase B thus increasing the amount of reactive ligands. Hexb^-/- ^DCs fail to generate iGb3 in the lysosome because they lack the β-hexosaminidase B required to remove the terminal GalNAc from iGb4 [35].

*In vitro*, exogenous addition of iGb3 to BMDCs aiming at iNKT cell activation was 100-fold less effective than α-GalCer as measured by IL-2 production. We found that iGb3 at 1 μg/ml or α-GalCer at 10 ng/ml activated NKT cells to produce 1 ng/ml of IL-2. Why should then studies on iGb3 be pursued on tumor cells apart from the fact that these isoglobohexosylceramides be endogenous constituents of these cells possibly in higher amounts than in normal cells? In B16F10-Nex2 cells iGb3 and iGb4 (precursor of iGb3) are natural effector molecules that may be shed in the microenvironment and thus be processed and presented by DCs to iNKT cells able to lyse tumor cells and to produce cytokines. This is an important component of the immunosurveillance in C57Bl/6 mice since CD1d-KO animals are significantly more susceptible to melanoma growth than WT mice. This effect is regulated by GM3 which is abundantly expressed at the tumor cell surface. In a melanoma metastatic model, however, we found that iGb3 (20 μg/ml)-loaded CD11c^+^CD1d^+ ^BMDCs reduced the number of lung nodules 4-fold, the same level of anti tumor protection obtained with α-GalCer at 200 ng/ml. Recognition that iGb3 can be protective against tumors when presented by DCs, suggests that a Th-1 immune response has been stimulated. Therefore iGb3 is an important mediator of iNKT cell activation which may contribute to host resistance to melanoma. To be used pharmacologically modifications in iGb3 structure are being investigated to improve its stimulatory activity. The iGb3 analog 4"'-dh-iGb3 (nonreducing terminal Gal deoxidized at 4-OH) and 4-OH-iGb3 (additional hydroxyl group on C4 of phytosphingosine) promoted significantly greater IFN-γ production [36]. Although 4"'-dh-iGb3 is still less potent than α-GalCer, the latter exerts such a strong activation of NKT cells with a single administration that they become anergic to α-GalCer restimulation for at least 30 days [37,38]. Moreover, α-GalCer may activate iNKT cells to produce both Th-1 and Th-2 cytokines thus limiting its therapeutic effectiveness. Chemical derivatives of α-Gal Cer with modified ceramides are being tested aiming at immune responses specifically directed to a Th-1 or Th-2 type [39].

It is conceivable that other components in the lipid fractions of B16F10-Nex2 melanoma may activate NKT cells. Such activity would be difficult to detect in a complex mixture containing inhibitory GM3 species. We looked at the neutral glycolipid fraction after removal of acidic species and besides the globo- and isoglobohexosides identified, we also detected several dihexosylceramide species with different ceramide composition (data not shown). Further fractionation and structural and functional analysis of the neutral glycolipid fraction and other B16F10-Nex2 melanoma-derived lipid fractions are necessary to explore this issue.

In conclusion, glycolipids iGb3, Gb3, iGb4 and Gb4 have been identified in murine melanoma cells. Our results demonstrate the important role of iNKT cells in the immune cellular protection against susceptible animals challenged with murine B16F10-Nex2 melanoma cells and show the activation of these cells by iGb3 and negative modulation by GM3. BMDCs primed with iGb3 protected against tumor development and metastasis and this effect depended on CD1d-restricted iNKTs. The present study stimulates further investigation on the use of iGb3 and derivatives in the immunopharmacology of melanoma and other tumors.

## Abbreviations

BMDC: bone marrow dendritic cells; WT: wild type; KO: knock-out; FBS: fetal bovine serum; Gb3 and iGb3: globo- and isoglobotrihexosylceramide; Gb4 and iGb4: globo- and isoglobotetrahexosylceramide; ESI-LIT-MS: electrospray ionization-linear ion trap-mass spectrometry; iNKT: invariant natural killer T cells; TCR: T cell receptor; HPTLC: high performance thin layer chromatography; GM3: monosialylglycosphingolipid (NANA-Hex-Hex-Cer); C-M-W: chloroform, methanol, water; GM-CSF: granulocyte-macrophage colony-stimulating factor.

## Competing interests

The authors declare that they have no competing interests.

## Authors' contributions

BRD designed and performed all biochemical and cell biological experiments, carried out data analysis and drafted the manuscript. EGR assisted in cell biological and immunological experiments, in vivo experiments and their design. LN assisted in the early lipid extractions and characterization as well as in the final draft of the manuscript. ESN and ICA extracted and purified glycosphingolipids, designed and carried out the ESI-LIT-MS experiments, elaborated MS figures, and wrote part of the manuscript (MS data). LRT, as Chairman of UNONEX conceived the study, coordinated its execution and design, and drafted and produced the final version of the manuscript. All authors read and approved the present version of the manuscript.
